# Supplementary material for: Impact of the COVID-19 pandemic on psychological distress and biological rhythm in China’s general population: A path analysis model
Source: PLoS One. 2022 Jul 8;17(7):e0271285. doi: 10.1371/journal.pone.0271285 (PMC9269873; doi:10.1371/journal.pone.0271285)
Supplement: S1 Table — (DOCX) [file pone.0271285.s001.docx]

**S1 Table.** Effect size of social-demographic information, psychological distress, social support, biological rhythm, and media information preference

|  | Cohen's d/  Cramer's Phi* | 95% Confidence Interval |
| --- | --- | --- |
| ***Age*** | -0.0445 | -0.1150~0.0260 |
| ***Education Level*** |  |  |
| Middle School or Below | 0.0484 | 0.0133~0.0757 |
| High School |  |  |
| Bachelor’s degree |  |  |
| Master’s degree or Above |  |  |
| ***Residence*** |  |  |
| Urban Areas | -0.0461 | -0.0807~-0.0114 |
| Rural Areas |  |  |
| ***Marital Status*** |  |  |
| Unmarried | 0.0517 | 0.0170~0.0862 |
| Married |  |  |
| ***Information Preference*** |  |  |
| Positive or Negative Info. | 0.0554 | 0.0211~0.0897 |
| Both of the Above |  |  |
| ***Social Support Total Scores*** | -0.1208 | -0.1914~-0.0503 |
| ***Somatization Scores*** | 0.1509 | 0.0803~0.2215 |
| ***Depression Scores*** | 0.0675 | -0.0030~0.1380 |
| ***Anxiety Scores*** | 0.0564 | -0.0141~0.1269 |
| ***Global Severity Index*** | 0.0942 | 0.0237~0.1648 |
| ***BRIAN Total Scores*** | -0.0668 | -.0.1373~0.0037 |

Note: * For continuous variables like age, social support total scores and other scales’ scores, we used Cohen's d to show the effect size. For categorical variables like education level, residence, marital status, and information preference, we used Cramer's Phi to show the effect size.
