# Supplementary material for: Impact of the COVID-19 pandemic on psychological distress and biological rhythm in China’s general population: A path analysis model
Source: PLoS One. 2022 Jul 8;17(7):e0271285. doi: 10.1371/journal.pone.0271285 (PMC9269873; doi:10.1371/journal.pone.0271285)
Supplement: S1 Appendix — (PDF) [file pone.0271285.s002.pdf]

知情同意书

今年的春节，一场突如其来的疫情打乱了我们的生活。手机、报纸、电视广播......无时无刻地向我们传递着这场疫情的动态。面对海量的媒体信息，你我也许会惊喜、会惆怅，因为家人、朋友的平安成为了我们最大的牵挂。

为了更好地了解在新冠疫情下普通民众的各项心理状况，及时有效地为广大群众提供心理援助，**四川大学华西医院心理卫生中心**发起本次匿名调查，各项问卷信息将按照相关法规实施绝对性的保密。

该研究调查对您目前及将来的健康不会有任何损害，我们恳请您能参加本调查。若您完成了本次问卷，即默认为您已签署知情同意。本次调查耗时约 8 分钟，感谢您的积极参与！

○了解以上信息，同意参加

○拒绝参加
