# Supplementary material for: Impact of the COVID-19 pandemic on psychological distress and biological rhythm in China’s general population: A path analysis model
Source: PLoS One. 2022 Jul 8;17(7):e0271285. doi: 10.1371/journal.pone.0271285 (PMC9269873; doi:10.1371/journal.pone.0271285)
Supplement: Attachment — Submitted filename: PONE-D-21-14840.pdf [file pone.0271285.s003.pdf]

# PLOS ONE

# Impact of the COVID-19 Pandemic on Psychological Distress and Biological Rhythm in China's General Population: A Path Analysis Model

--Manuscript Draft--

| | |
|---|---|
| **Manuscript Number:** | PONE-D-21-14840 |
| **Article Type:** | Research Article |
| **Full Title:** | Impact of the COVID-19 Pandemic on Psychological Distress and Biological Rhythm in China's General Population: A Path Analysis Model |
| **Short Title:** | Impact of the COVID-19 Pandemic on Psychological Distress and Biological Rhythm in China's General Population |
| **Corresponding Author:** | Xiaohong Ma, Ph.D<br>The Psychiatric Laboratory & the Department of Psychiatry<br>Chengdu, CHINA |
| **Keywords:** | psychological distress; Gender; Social support; Biological rhythm; Information preference |
| **Abstract:** | Background : When facing major emergency public accidents, males and females may react differently. Our research aimed to assess the influence of gender difference on social support, information preference, biological rhythm, psychological distress, and the possible interaction among these factors during the COVID-19 pandemic.<br>Methods: In this cross-sectional study, 3,237 respondents aged 12 years and older finished the online survey. Levels of social support, information preference, biological rhythm, and psychological distress were assessed using validated scales. A path analysis was conducted to explore the possible association among these variables.<br>Results: The path analysis indicated that females with high levels of social support had a lower possibility of biological rhythm disorder and lower levels of somatization symptoms of psychological distress during the COVID-19 pandemic. The influence of social support on somatization symptoms was exerted via biological rhythm. Females tended to believe both negative information and positive information, while males preferred to access more extreme information.<br>Conclusion: Our results highlighted gender difference in study variables during the COVID-19 pandemic and the importance of social support in alleviating psychological distress and biological rhythm disorders. Moreover, we confirmed that information preference differ significantly by somatization symptoms of psychological distress, suggesting more efforts to provide more individualized epidemic information. Longitudinal research is required to further explore casual inferences. |
| **Order of Authors:** | YiKai Dou |
| | Huanhuan Fan |
| | Xiao Yang |
| | Yue Du |
| | Yu Wang |
| | Min Wang |
| | Zijian Zhang |
| | Xiongwei Qi |
| | Yuling Luo |
| | Ruiqing Luo |
| | Xiaohong Ma, Ph.D |
| **Additional Information:** | |
| **Question** | **Response** |
| **Financial Disclosure** | The author received no specific funding for this work. |

*Powered by Editorial Manager® and ProduXion Manager® from Aries Systems Corporation*

| | |
|---|---|
| Enter a financial disclosure statement that describes the sources of funding for the work included in this submission. Review the submission guidelines for detailed requirements. View published research articles from *PLOS ONE* for specific examples.<br><br>This statement is required for submission and **will appear in the published article** if the submission is accepted. Please make sure it is accurate.<br><br>**Unfunded studies**<br>Enter: *The author(s) received no specific funding for this work.*<br><br>**Funded studies**<br>Enter a statement with the following details:<br>• Initials of the authors who received each award<br>• Grant numbers awarded to each author<br>• The full name of each funder<br>• URL of each funder website<br>• Did the sponsors or funders play any role in the study design, data collection and analysis, decision to publish, or preparation of the manuscript?<br>• **NO** - Include this sentence at the end of your statement: *The funders had no role in study design, data collection and analysis, decision to publish, or preparation of the manuscript.*<br>• **YES** - Specify the role(s) played.<br><br>* typeset | |
| **Competing Interests**<br><br>Use the instructions below to enter a competing interest statement for this submission. On behalf of all authors, disclose any competing interests that could be perceived to bias this work—acknowledging all financial support and any other relevant financial or non-financial competing interests.<br><br>This statement is required for submission and **will appear in the published article** if | The authors have declared that no competing interests exist. |

Powered by Editorial Manager® and ProduXion Manager® from Aries Systems Corporation

| | |
|---|---|
| the submission is accepted. Please make sure it is accurate and that any funding sources listed in your Funding Information later in the submission form are also declared in your Financial Disclosure statement.<br><br>View published research articles from *PLOS ONE* for specific examples.<br><br>**NO authors have competing interests**<br><br>Enter: *The authors have declared that no competing interests exist.*<br><br>**Authors with competing interests**<br><br>Enter competing interest details beginning with this statement:<br><br>*I have read the journal's policy and the authors of this manuscript have the following competing interests: [insert competing interests here]*<br><br>* typeset | |
| **Ethics Statement**<br><br>Enter an ethics statement for this submission. This statement is required if the study involved:<br><br>• Human participants<br>• Human specimens or tissue<br>• Vertebrate animals or cephalopods<br>• Vertebrate embryos or tissues<br>• Field research<br><br>Write "N/A" if the submission does not require an ethics statement.<br><br>General guidance is provided below. Consult the submission guidelines for detailed instructions. **Make sure that all information entered here is included in the Methods section of the manuscript.** | Informed consent was acquired before each participant decided to finish this questionnaire. To protect the privacy of participants, all collected information was anonymous. This research was approved by the Ethics Committee of West China Hospital of Sichuan University(No.2020-178). |

| | |
|---|---|
| **Format for specific study types**<br><br>**Human Subject Research (involving human participants and/or tissue)**<br>• Give the name of the institutional review board or ethics committee that approved the study<br>• Include the approval number and/or a statement indicating approval of this research<br>• Indicate the form of consent obtained (written/oral) or the reason that consent was not obtained (e.g. the data were analyzed anonymously)<br><br>**Animal Research (involving vertebrate animals, embryos or tissues)**<br>• Provide the name of the Institutional Animal Care and Use Committee (IACUC) or other relevant ethics board that reviewed the study protocol, and indicate whether they approved this research or granted a formal waiver of ethical approval<br>• Include an approval number if one was obtained<br>• If the study involved *non-human primates*, add *additional details* about animal welfare and steps taken to ameliorate suffering<br>• If anesthesia, euthanasia, or any kind of animal sacrifice is part of the study, include briefly which substances and/or methods were applied<br><br>**Field Research**<br><br>Include the following details if this study involves the collection of plant, animal, or other materials from a natural setting:<br>• Field permit number<br>• Name of the institution or relevant body that granted permission | |
| **Data Availability**<br><br>Authors are required to make all data underlying the findings described fully available, without restriction, and from the time of publication. PLOS allows rare exceptions to address legal and ethical concerns. See the PLOS Data Policy and FAQ for detailed information. | No - some restrictions will apply |

Powered by Editorial Manager® and ProduXion Manager® from Aries Systems Corporation

| | |
|---|---|
| A Data Availability Statement describing where the data can be found is required at submission. Your answers to this question constitute the Data Availability Statement and **will be published in the article**, if accepted.<br><br>**Important:** Stating 'data available on request from the author' is not sufficient. If your data are only available upon request, select 'No' for the first question and explain your exceptional situation in the text box.<br><br>Do the authors confirm that all data underlying the findings described in their manuscript are fully available without restriction? | |
| **Describe where the data may be found in full sentences. If you are copying our sample text, replace any instances of XXX with the appropriate details.**<br><br>• If the data are **held or will be held in a public repository**, include URLs, accession numbers or DOIs. If this information will only be available after acceptance, indicate this by ticking the box below. For example: *All XXX files are available from the XXX database (accession number(s) XXX, XXX.*).<br>• If the data are all contained **within the manuscript and/or Supporting Information files**, enter the following: *All relevant data are within the manuscript and its Supporting Information files.*<br>• If neither of these applies but you are able to provide **details of access elsewhere**, with or without limitations, please do so. For example:<br><br>*Data cannot be shared publicly because of [XXX]. Data are available from the XXX Institutional Data Access / Ethics Committee (contact via XXX) for researchers who meet the criteria for access to confidential data.*<br><br>*The data underlying the results presented in the study are available from (include the name of the third party* | Data are available from the West China Hospital Institutional Data Access / Ethics Committee (contact via maxiaohong@scu.edu.cn) for researchers who meet the criteria for access to confidential data |

| | |
|---|---|
| *and contact information or URL).*<br>• This text is appropriate if the data are owned by a third party and authors do not have permission to share the data.<br><br>* typeset | |
| Additional data availability information: | |

Powered by Editorial Manager® and ProduXion Manager® from Aries Systems Corporation

Manuscript Click here to access/download;Manuscript;Manuscript.docx

# Impact of the COVID-19 Pandemic on Psychological Distress and Biological Rhythm in China's General Population: A Path Analysis Model

Yikai Dou[1] · Huanhuan Fan[1] · Xiao Yang[1] · Yue Du[1] · Yu Wang[1] · Min Wang[1] · Zijian Zhang[1] · Xiongwei Qi[1] · Yuling Luo[1] · Ruiqing Luo[1] · Xiaohong Ma[123]*

**Affiliations:**

1. Psychiatric Laboratory and Mental Health Center, West China Hospital of Sichuan University, Chengdu, Sichuan, 610064, China.

2. Psychiatric Laboratory and Mental Health Center, the State Key Laboratory of Biotherapy, West China Hospital of Sichuan University, Chengdu, 610041, China.

3. West China Brain Research Center, West China Hospital of Sichuan University, Chengdu, China.

*__Corresponding author E-mail__: maxiaohong@scu.edu.cn

## Abstract

**Background:** When facing major emergency public accidents, males and females may react differently. Our research aimed to assess the influence of gender difference on social support, information preference, biological rhythm, psychological distress, and the possible interaction among these factors during the COVID-19 pandemic.

**Methods:** In this cross-sectional study, 3,237 respondents aged 12 years and older finished the online survey. Levels of social support, information preference, biological rhythm, and psychological distress were assessed using validated scales. A path analysis was conducted to explore the possible association among these variables.

**Results:** The path analysis indicated that females with high levels of social support had a lower possibility of biological rhythm disorder and lower levels of somatization symptoms of psychological distress during the COVID-19 pandemic. The influence of social support on somatization symptoms was exerted via biological rhythm. Females tended to believe both negative information and positive information, while males preferred to access more extreme information.

**Conclusion:** Our results highlighted gender difference in study variables during the COVID-19 pandemic and the importance of social support in alleviating psychological distress and biological rhythm disorders. Moreover, we confirmed that information preference differ significantly by somatization symptoms of psychological distress, suggesting more efforts to provide more individualized epidemic information. Longitudinal research is required to further explore casual inferences.

## Keywords

Psychological distress; Gender; Social support; Biological rhythm; Information preference

## Introduction

Since the outbreak of the novel coronavirus disease 2019 (COVID-19) in late December, 2019, it has spread rapidly throughout almost all regions in the world[1]. Exceeding 153,000,000 people were confirmed to be infected with this severe respiratory infectious disease, and more than 216 countries or territories were affected [2]. COVID-19 presents an urgent and vital threat to global public health and social economy [3, 4]. In many countries and regions, governments asked residents to reduce unnecessary outdoor activities, and shopping malls and public transportations were also closed to avoid

intimidate contact. Social distancing, self-isolation and travel restrictions have led to a reduced workforce across all economic sectors and caused many jobs to be lost. Even the Olympic Games that are held every four years have been postponed. As an internationally concerned public health emergency, the COVID-19 pandemic has been influencing our regular lifestyle greatly and has a wide range of adverse psychological impacts on the general population [5-8].

Previous studies have shown that individuals may go through fear of being infected or even of death themselves, feeling hopeless or helpless and even ashamed once been infected [9]. Approximately 10%-30% general population were much concerned with being infected when an influenza outbreak occurred [10]. Meanwhile, self-isolation at home can also cause high prevalence of symptoms of psychological distress such as insomnia, stress, emotional disturbance, and other psychological disorders [11]. Therefore, individual social support during home quarantine and accurate, timely and effective epidemic information is vital for the general public. The elderly, the single and others who also live alone have to face the horrible infectious disease without family members' company. Insufficient medical supplies such as face masks and disinfectants at the beginning of the COVID-19 pandemic increased fear and uncertainty brought about by this severe viral infection[12].

Furthermore , social media of unreliable sources usually provide much ambiguous epidemic information, and overloaded information may cause psychological distress in turn [13]. Effective risk information communication among people can reduce negative psychological responses and strong social support may play a role [14]. In addition, information preference can be essential in helping shape the public's risk perception and has been reported to be influenced by people's gender, age, social status etc. [15, 16] Therefore, information preference should be considered in the analysis of underlying influencing factors of risk perceptions of infectious diseases like COVID-19.

Besides social support and epidemic information preference, biological rhythm is another significant factor. Travel restrictions or home isolation disturbs the circadian rhythms. Staying up late, getting up late and lying in bed during non-sleeping time all decrease activity and meal frequency, causing rhythm disorders in eating, sleeping, social activities etc. and aggravating people's physical and psychological distress in the meantime.

Therefore, we conducted this study during the COVID-19 pandemic in an attempt to identify a possible relationship of gender difference with social support, biological rhythm, information preference, and psychological distress. This is the first study to examine all these factors together in China's general population during the COVID-19 pandemic.

# Methods

## Study Design and Participants

A cross-sectional online survey was conducted via Chinese social applications (APPs) WeChat and Weibo, the Chinese equivalent of Twitter, in China's mainland between 26 February, 2020 and 2 March, 2020. During this period most people were still isolated at home because of the COVID-19 pandemic. Participants would be excluded if they under 12 years old or not living in China's mainland. This survey contained demographic information such as age, gender, education level, and social status, and took approximately 10-15 minutes for each participant to complete. Other vital information including social support, biological rhythm, media information preference, and psychological distress were also assessed. Informed consent was acquired before each participant decided to finish this questionnaire. To protect the privacy of participants, all collected information was anonymous. This research was

approved by the Ethics Committee of West China Hospital of Sichuan University.

### Measure Instrument

#### *Brief Symptom Inventory-18 (BSI-18)*

BSI-18 is a self-report symptoms checklist which is commonly used to evaluate psychological distress of respondents in the past one week [17]. It contains 18 items and can be divided into three subscales (somatization, depression, and anxiety). Scores of each item in this five-point Likert scale range from 0 (not at all) to 4 (very much). The total score of BSI-18 is also called "global severity index (GSI)". The Cronbach's alpha equals to 0.98, 0.94, 0.93, and 0.95 for GSI, somatization, depression, and anxiety, respectively, suggesting a good internal consistency reliability for our research sample. The Chinese version of BSI-18 has been used among China's patients and general population [18-20].

#### *Social Support Rating Scale (SSRS)*

SSRS was used for the measurement of social support. It has been widely applied in different psychological studies; and its Chinese version was developed by Professor Xiao in 1998 [21]. The SSRS consists of 10 items, and 3 dimensions of social support were evaluated, including subjective support (4 items), objective support (3 items), and support utilization (3 items). Scores of three subscales were simply added up, generating a social support total score ranging from 12 to 66. High scores demonstrate having a higher level of social support received by the respondents [22, 23]. In our research sample, the Cronbach's alpha of total support scores was 0.62, indicating a moderate reliability.

#### *Biological Rhythm Interview of Assessment in Neuropsychiatry (BRIAN)*

BRIAN was applied to assess the degree of biological rhythm dysregulation. This four-point scale contains 21 items. Four primary domains of rhythm disturbance, involving sleep (5 items), social rhythm (5 items), activity (4 items), and eating pattern (4 items) were evaluated. Another domain referring to chronotype was not taken into consideration in the total BRIAN score[24]. Higher total scores signify strong disturbance of biological rhythm. Previous studies show that BRIAN has good psychometric properties in patients with mood disorder or general school students [25, 26]. The scale has been translated into different versions. The Cronbach's alpha of total BRIAN scores in our sample was 0.95, indicating a good reliability[27].

#### *Media Information Preference*

Respondents' attitude toward media information was measured using one question: "Which kind of information do you usually pay attention to?" Two choices were provided: 1) Either negative media information or positive media information ; and 2 ) Both negative and positive media information. This question was designed based on some previous researches which aimed to reflect the preference of different respondents for the magnanimity of media information on cellphone social APPs or television[13, 16, 28, 29].

### Statistical Analysis

Data analysis was performed using Stata/SE 15.1 software. First, for continuous variables such as age and scores of psychological distress, *t* test was used to assess the statistical significance; for categorical variables, $x^2$ test was used to describe the constituent ratio of education level, media information preference etc. Second, correlations between gender, media information preference, social support total scores, somatization scores, depression scores, anxiety scores, global severity index, and BRAIN total scores were calculated using Spearman's rank correlation coefficients. Finally, aiming to study the possible causal relationship, we constructed a structural equation model (SEM) and applied path analysis to testify the relationship among interrelated study variables in a hypothesized model. In our SEM, somatization scores were modeled as outcome variables, while gender was modeled as an observed variable, and social support total scores, media information preference, and biological rhythm were modeled as mediators. SEM estimated both the direct and indirect effects one variable had on the outcome variable. Several indices were used to determine whether the hypothesized model fit the observed data. The chi-square value was the original fit index for structural equation models. An acceptable model means $P < 0.05$ in the chi-square. However, some previous studies shows that the chi-square test is so sensitive to sample size that it always rejects the SEM especially when large samples are used[30]. Thus, several alternative fit indices were included in our study. Absolute fit indices such as the Root Mean Square Error of Approximation (RMSEA), the Standardized Root Mean Square Residual (SRMR), and the Goodness of Fit Index (GFI) were chosen to evaluate the structural model. It would be considered as a good model if RMSEA <0.08, SRMR <0.08, and GFI >0.90. Besides, incremental fit indices such as Tucker Lewis Index (TLI) and Comparative Fit Index (CFI) were also proposed. Values above 0.90 for TLI and CFI were considered an acceptable fit. Statistical significance was accepted at $P < 0.05$.

## Results

### Description of the sample

A total of 3,246 respondents registered in our questionnaire. In the end, 9 were excluded because they were below 12 years (n=8) or did not live in China's mainland (n=1). Their social-demographic information is shown in Table 1. Male (n=1,277) and female (n=1,960) differed significantly in residence ($x^2$=6.87), marital status ($x^2$=8.64), and information preference($x^2$=9.92); the corresponding P values were 0.0090, 0.0030, and 0.0020, respectively. The two groups did not differ significantly in age (t=-1.23; p=0.2162) or education level ($x^2$=7.58; p=0.0560). Social support total scores, psychological distress scores, and BRIAN total scores were compared (Table 1). The mean scores of social support were obviously higher in female than in male (p=0.0008). Psychological distress scores differed significantly in somatization scores ($P < 0.0001$) and global severity index (P=0.0088) between the two groups. The two groups did not differ significantly in depression scores, anxiety scores, or BRIAN total scores.

**Table 1** Gender Difference in Social-demographic Information, Psychological Distress, Social Support, Biological Rhythm, and Media Information Preference

| | Male (N=1277) | Female (N=1960) | T/$X^2$ Value | P Value |
|---|---|---|---|---|
| ***Age*** | 30.70±9.36 | 31.12±9.56 | -1.23 | 0.2162 |
| ***Education Level*** | | | | |

| | | | | |
|---|---|---|---|---|
| Middle School or Below | 122 | 136 | 7.58 | 0.0560 |
| High School | 148 | 221 | | |
| Bachelor’s degree | 771 | 1221 | | |
| Master’s degree or Above | 236 | 382 | | |
| ***Residence*** | | | | |
| Urban Areas | 768 | 1268 | 6.87 | 0.0090 |
| Rural Areas | 509 | 692 | | |
| ***Marital Status*** | | | | |
| Unmarried | 661 | 911 | 8.64 | 0.0030 |
| Married | 616 | 1049 | | |
| ***Information Preference*** | | | | |
| Positive or Negative Info. | 614 | 832 | 9.92 | 0.0020 |
| Both of the Above | 663 | 1128 | | |
| ***Social Support Total Scores*** | 37.57±8,26 | 38.54±7.89 | -3.36 | 0.0008 |
| ***Somatization Scores*** | 9.98±6.21 | 9.13±5.19 | 4.20 | <0.0001 |
| ***Depression Scores*** | 10.84±6.34 | 10.44±5.62 | 1.88 | 0.0606 |
| ***Anxiety Scores*** | 10.4±6.29 | 10.06±5.71 | 1.57 | 0.1170 |
| ***Global Severity Index*** | 31.22±18.37 | 29.64±15.66 | 2.62 | 0.0088 |
| ***BRIAN Total Scores*** | 31.27±12.58 | 32.07±11.54 | -1.86 | 0.0633 |

**Correlations among Study Variables**

The correlations between study variables were studied (Table 2). Obviously, gender had a positive correlation with media information preference(r=0.0554), social support scores(r=0.0546), and BRIAN total scores(r=0.0517). However, it had a negative correlation with somatization scores(r=-.00380). Besides, media information preference had a negative correlation with all study variables except BRIAN total scores. In addition, social support scores showed a negative correlation with both psychological distress scores and BRIAN total scores.

**Table 2**. Spearman correlations among study variables ( N=3237)

| Variables | **1** | **2** | **3** | **4** | **5** | **6** | **7** | **8** |
|---|---|---|---|---|---|---|---|---|
| **1. Gender** | — | | | | | | | |
| **2. Info. Preference** | 0.0554$^{*}$ | — | | | | | | |
| **3. Support** | 0.0546$^{*}$ | -0.1105$^{**}$ | — | | | | | |
| **4. SOM** | -0.0380$^{*}$ | -0.0834$^{**}$ | -0.1689$^{**}$ | — | | | | |
| **5. DEP** | -0.0016 | -0.0366$^{*}$ | -0.2535$^{**}$ | 0.7801$^{**}$ | — | | | |
| **6. ANX** | 0.0046 | -0.0731$^{**}$ | -0.1694$^{**}$ | 0.7962$^{**}$ | 0.8490$^{**}$ | — | | |
| **7. GSI** | 0.0032 | -0.0483$^{*}$ | -0.2181$^{**}$ | 0.8648$^{**}$ | 0.9543$^{**}$ | 0.9285$^{**}$ | — | |
| **8. BRIAN** | 0.0517$^{*}$ | 0.0515$^{*}$ | -0.2381$^{**}$ | 0.6212$^{**}$ | 0.6882$^{**}$ | 0.6491$^{**}$ | 0.7017$^{**}$ | — |

Note：(1) **Support:** Social Support Total Scores; **SOM:** Somatization Scores; **DEP:** Depression Scores; **ANX:** Anxiety Scores; **GSI:** Global Severity Index; **BRIAN:** BRAIN Total Scores

(2) *: P < 0.05 , **: P < 0.001

**Path Analysis of the Hypothesized Model**

According to correlations among study variables, we built a SEM to explore the possible causal relationship of study variables, and the fit dices were depicted (Table 3). The initial hypothesized path model fit the data poorly, and $X^2$ could not be calculated. Therefore, we had to sequentially remove three original pathways to generate the modified model (Table 3). Model 2 and Model 3 were invalid for Tucker-Lewis index more than 1. Moreover, even Model 4 fit all indices all in a reasonable range, P values were insignificant in two pathways after subsequent direct effects analysis (Table 4).

**Table 3**. Path Analysis Steps with Fit Indices

| | $X^2$ | **P Value*** | **RMSEA*** | **CFI*** | **TLI*** | **SRMR*** |
|---|---|---|---|---|---|---|
| **1. Hypothesized Model** | 0.0000 | — | 0.0000 | 1.0000 | 1.0000 | 0.0000 |
| **2. Support→SOM*** | 0.5190 | 0.4710 | <0.001 | 1.0000 | 1.0020 | 0.0020 |
| **3. Info→Biorhythm*** | 0.0250 | 0.8740 | <0.001 | 1.0000 | 1.0040 | 0.0010 |
| **4. Gender→Biorhythm*** | 7.4650 | 0.0060 | 0.045 | 0.9970 | 0.9720 | 0.0150 |
| **5. Modified Model** | 7.9800 | 0.0463 | 0.0230 | 0.9980 | 0.9930 | 0.0150 |

Note:

**P value**: Chi $^2$ Test for Model vs. saturated; **RMSEA**: Root mean squared error of approximation

**CFI**: Comparative fit index; **TLI**: Tucker-Lewis index; **SRMR**: Standardized root mean squared residual

**Support→SOM**: Removing pathway between social support and somatization scores, same as **Info→Biorhythm** and **Gneder→Biorhythm**

**Table 4**. Direct Effects in Model 4

| | **Coefficient** | **SE** | **Z Value** | **P value** | **CI** |
|---|---|---|---|---|---|
| **Gender→Support** | 0.9715 | 0.2891 | 3.36 | 0.001 | 0.4050~1.5380 |
| **Gender→Info** | 0.0628 | 0.0178 | 3.53 | <0.001 | 0.0280~0.0976 |
| **Support→Info** | -0.0066 | 0.0010 | -6.15 | <0.001 | -0.0088~-0.0045 |
| **Gender→BioRhythm** | | | No Path | | |
| **Info→BioRhythm** | 0.0042 | 0.4134 | 0.01 | 0.9920 | -0.8061~0.8145 |
| **Support→BioRhythm** | -0.3489 | 0.0255 | -13.67 | <0.001 | -0.3989~-0.2988 |
| **Gender→SOM** | -0.9990 | 0.1475 | -6.77 | <0.001 | -1.2880~-0.7099 |
| **BioRhythm→SOM** | 0.3153 | 0.0062 | 51.07 | <0.001 | 0.3032~0.3274 |
| **Info→SOM** | -1.6661 | 0.1454 | -11.46 | <0.001 | -1.9510~-1.3811 |
| **Support→SOM** | -0.0067 | 0.0092 | -0.72 | 0.4710 | -0.0248~0.0115 |

The modified model had good fit indices (Fig. 1). Gender had a direct influence on social support, information preference, and somatization scores of psychological distress. Besides, somatization scores can be directly predicted by gender, information preference, and biological rhythm. In addition, it can

be indirectly predicted by social support.

***Influence of Gender on Endogenous Variables and Outcome Variable***

Gender difference had direct associations with social support, information preference, and somatization symptoms of psychological distress. Female was associated with a higher level of social support (β=0.9715, P=0.001) and lower somatization scores of psychological distress (β=-1.0007, P<0.001). Male (β=0.0628, P<0.001) was associated with more extreme media information. Neither gender nor information preference had any direct influence on biological rhythm.

***Influence of Endogenous Variables on Outcome Variable***

Social support was directly associated with biological rhythm (β=-0.3489, P < 0.001) and information preference (β=-0.0066, P < 0.001). The influence of social support on somatization symptoms was exerted through the process variables: biological rhythm. Lower biological rhythm total scores were associated with a lower level of somatization symptoms of psychological distress (β=-1.0007, P < 0.001). For example, male might predict a lower level of social support, while weaker social support was associated with biological rhythm disorder, which further predicted higher scores of somatization symptoms. Besides, female might prefer to choose both negative and positive information, while male preferred to access more extreme information instead (Table 5).

**Figure 1**. Modified structural equation model. Standardized beta coefficients are noted above each path. Solid lines indicate significant pathways, and perforated lines represent pathways removed from hypothesized model. Model fit indices: $X^2$=7.98 (P= 0.0463), Tucker-Lewis index = 0.9930, comparative fit index = 0.9980, root-mean-square error of approximation = 0.0230, and standardized root-mean-square residual = 0.0150.

Table 5. Direct effects and indirect effects in modified model

| | Direct Effects | | | | | Indirect Effects | | | | |
|---|---|---|---|---|---|---|---|---|---|---|
| | Coefficient | SE | Z Value | P Value | CI | Coefficient | SE | Z Value | P Value | CI |
| Gender→Support | 0.9715 | 0.2891 | 3.36 | 0.001 | 0.4049~1.5380 | No Path | | | | |
| Gender→Info | 0.0628 | 0.0178 | 3.53 | <0.001 | 0.0279~0.0976 | -0.0064 | 0.0022 | -2.95 | 0.003 | -0.0107~-0.0022 |
| Support→Info | -0.0066 | 0.0011 | -6.15 | <0.001 | -0.0088~-0.0045 | No Path | | | | |
| Gender→BioRhythm | No Path | | | | | -0.3389 | 0.1038 | -3.26 | 0.001 | -0.5424~-0.1355 |
| Support→BioRhythm | -0.3489 | 0.0254 | -13.89 | <0.001 | -0.3987~-0.2991 | No Path | | | | |
| Gender→SOM | -1.0007 | 0.1471 | -6.85 | <0.001 | -1.2952~-0.7186 | -0.2005 | 0.0426 | -4.70 | <0.001 | -0.2841~-0.1168 |
| BioRhythm→SOM | 0.3164 | 0.0060 | 52.73 | <0.001 | 0.3046~0.3282 | No Path | | | | |
| Info→SOM | -1.6651 | 0.1446 | -11.45 | <0.001 | -1.9384~-1.3717 | No Path | | | | |
| Support→SOM | No Path | | | | | -0.0994 | 0.0085 | -11.64 | <0.001 | -0.1161~-0.0827 |

## Discussion

In this cross-sectional study involving 3237 participants, we found significant difference in social total scores and global severity index, which goes in line with the existing literature [31-33]. Nevertheless, most previous studies reported that females suffer from more somatization symptoms than males and are more vulnerable to psychological distress [32, 34].

The most important objective of our study was to explain the possible associations of social support, information preference, and biological rhythm between gender and somatization symptoms. In our study, path analysis indicated that females had a higher level of social support, suggesting a smaller possibility of biological rhythm disorder and a lower level of somatization symptoms of psychological distress against the background of COVID-19 pandemic. The direct effect of gender difference on somatization symptoms was also statistically significant. This finding was partly in accordance with previous studies that confirmed the positive function of social support to relieve psychological distress, especially in chronic disease or traumatic natural disaster accidents [35-37]. In our model, however, female was a protective factor, which is different from other study results. Extant studies showed that in female, the prevalence of psychological distress was higher and somatization symptoms were more obvious. Our findings may provide several explanations. First, female in general may access more easily sufficient social support from family members, collogues and / or friends[38, 39]. This means that females have more channels to obtain information about the COVID-19 pandemic. In addition, such communication can help them discern false, fake, or stigmatization epidemic information, which further relieves anxiety and somatization symptoms[40]. Second, for married men, home quarantine forced them to stay with their family in a sense, which very likely increased conflicts between the husband and wife due to limited recreational activities and personal space at home. In the context of the Chinese culture, most men are unwilling to tell their inner dissatisfaction to their intimate life partner, and it is difficult for them to express their inner anxious emotions, which might also increase their physical symptoms of psychological distress[41-43]. At the same time, Chinese fathers who have been largely absent in children’s education [44]had to spend more time and energy in taking care of and educating their children during self-isolation due to COVID-19, which could also augment negative psychological feelings. Third，for unmarried or single men who lived alone, self-isolation may be a big challenge because of insufficient social support and limited ways of expression. They may easily be confused by epidemic information and experience increased fear of pandemic COVID-19. Therefore, enhancement of social support among males during COVID-19 home isolation is critical for alleviating their somatization symptoms of psychological distress[45].

In addition，our path analysis suggested that to alleviate somatization symptoms among males, we need to fortify their social support in addition to correcting their dysfunctioning biological rhythm such as insomnia, eating pattern disorder, or daily circadian social activities. Social support was not directly associated with somatization symptoms; and biological rhythm as a mediator played an important role in relieving somatization symptoms of psychological distress. Our findings are in line with previous study results that psychological distress is associated with disruptions in sleep and circadian rhythm[46]. During home isolation people may stay up late unconsciously and have difficulty getting up the next morning, which could affect their daily eating pattern[47]. Furthermore, due to reduced outdoor physical activities, sleep problems and disturbance of the eating rhythm may also be aggravated.

Finally, we found that preference of extreme information also increased somatization scores of psychological distress, and that the male group preferred to choose either negative information or positive information. Gender difference indeed affected public information preference and their extent of risk perception. Related health-seeking behavior could also be influenced by gender. Females are more capable of perceiving risks and thus will be more proactive in taking related health-seeking

behaviors to weaken the negative impact of epidemic information[48, 49]. Stronger social support in the female group can guarantee effective interpersonal information exchanges, which may contribute to confirming the reliability of epidemic information[14]. Therefore , although gender difference has been observed in the access to epidemic information, the sufficient social support and varieties of communication channels that females could obtain partly compensated for the inadequacy in the access to media information[16].Therefore, females tend to be in a more neutral position when facing epidemic information of various kinds. These results have been partly confirmed by previous studies. The preference for specific information may be key determinants of the individual's perception of risk regarding the COVID-19 pandemic. The "24-hour a day, 7 days a week" exposure to intensive and extensive media coverage of the COVID-19 pandemic amplifies risk perception and fear, making the general public anxious in the face of uncertainty. Such uncertainty greatly increases the individual's psychological burden[50]. In addition, individuals differ largely in risk perception by educational backgrounds and knowledge levels [51]. Hence, as Wong L P emphasized, the dissemination of epidemic information should be dedicated to meeting the information needs of diverse sociodemographic and ethnic groups [16, 52]. Therefore, for different gender groups, the provision of epidemic information should also be tailored to individual needs.

## Limitations

The present study has three major limitations. First ,because of the cross-sectional nature of the study, the relationships among study variables demonstrated in the structured model were based on strong theoretical rationales. Future research is needed to further employ longitudinal panel data to better understand causal inferences among gender, social support, information preference, biological rhythm, and psychological distress. Second, all scales used in the present study are self-rating questionnaires. Self-reported bias was thus inevitable due to personal attitudes. Finally, most respondents did not come from high-risk areas like Hubei Province. Because the threat for COVID-19 and the corresponding psychological reaction may differ by risk areas.

## Conclusion

The study is the first step to uncover the direct and indirect effects of gender on somatization symptoms of psychological distress during the COVOD-19 pandemic, while social support, biological rhythm and information preference can be used as possible mediators. These findings highlight the gender difference in study variables during the COVID-19 pandemic and the importance of social support in alleviating psychological distress and biological rhythm disorders. Moreover, the influence of information preference on somatization symptoms of psychological distress differs greatly by gender, and public health policy-makers and mass media need to provide better-targeted epidemic information to different individuals.

## References


1. Ge, H., et al., *The epidemiology and clinical information about COVID-19.* European journal of clinical microbiology & infectious diseases : official publication of the European Society of Clinical Microbiology, 2020. **39**(6): p. 1011-1019.
2. Palacios Cruz, M., et al., *COVID-19, a worldwide public health emergency.* Revista clinica

espanola, 2020.
3. Contini, C., et al., *The novel zoonotic COVID-19 pandemic: An expected global health concern.* Journal of infection in developing countries, 2020. **14**(3): p. 254-264.
4. Lai, C.-C., et al., *Severe acute respiratory syndrome coronavirus 2 (SARS-CoV-2) and coronavirus disease-2019 (COVID-19): The epidemic and the challenges.* International journal of antimicrobial agents, 2020. **55**(3): p. 105924.
5. Sohrabi, C., et al., *World Health Organization declares global emergency: A review of the 2019 novel coronavirus (COVID-19).* International journal of surgery (London, England), 2020. **76**: p. 71-76.
6. Yahya, A.S., S. Khawaja, and J. Chukwuma, *The Impact of COVID-19 in Psychiatry.* The primary care companion for CNS disorders, 2020. **22**(2).
7. Ahmed, M.Z., et al., *Epidemic of COVID-19 in China and associated Psychological Problems.* Asian journal of psychiatry, 2020. **51**: p. 102092.
8. Torales, J., et al., *The outbreak of COVID-19 coronavirus and its impact on global mental health.* The International journal of social psychiatry, 2020: p. 20764020915212.
9. Wang, C., et al., *Immediate Psychological Responses and Associated Factors during the Initial Stage of the 2019 Coronavirus Disease (COVID-19) Epidemic among the General Population in China.* International journal of environmental research and public health, 2020. **17**(5).
10. Rubin, G.J., H.W.W. Potts, and S. Michie, *The impact of communications about swine flu (influenza A H1N1v) on public responses to the outbreak: results from 36 national telephone surveys in the UK.* Health technology assessment (Winchester, England), 2010. **14**(34): p. 183-266.
11. Brooks, S.K., et al., *The psychological impact of quarantine and how to reduce it: rapid review of the evidence.* Lancet (London, England), 2020. **395**(10227): p. 912-920.
12. Jakovljevic, M., et al., *COVID-19 Pandemia and Public and Global Mental Health from the Perspective of Global Health Securit.* Psychiatr Danub, 2020. **32**(1): p. 6-14.
13. Gao, J., et al., *Mental health problems and social media exposure during COVID-19 outbreak.* PloS one, 2020. **15**(4): p. e0231924.
14. Han, G. and Y. Liu, *Does Information Pattern Affect Risk Perception of Food Safety? A National Survey in China.* International journal of environmental research and public health, 2018. **15**(9).
15. Fang, D., et al., *Relationships among trust in messages, risk perception, and risk reduction preferences based upon avian influenza in Taiwan.* International journal of environmental research and public health, 2012. **9**(8): p. 2742-2757.
16. Wong, L.P. and I.C. Sam, *Public sources of information and information needs for pandemic influenza A(H1N1).* Journal of community health, 2010. **35**(6): p. 676-682.
17. Meijer, R.R., R.M. de Vries, and V. van Bruggen, *An evaluation of the Brief Symptom Inventory-18 using item response theory: which items are most strongly related to psychological distress?* Psychological assessment, 2011. **23**(1): p. 193-202.
18. Merport, A., et al., *Can the distress thermometer (DT) identify significant psychological distress in long-term cancer survivors? A comparison with the Brief Symptom Inventory-18 (BSI-18).* Supportive care in cancer : official journal of the Multinational Association of Supportive Care in Cancer, 2012. **20**(1): p. 195-198.

19. Wang, J., et al., *Factorial structure of the Brief Symptom Inventory (BSI)-18 among Chinese drug users.* Drug and alcohol dependence, 2013. **133**(2): p. 368-375.
20. Li, M., et al., *Psychometric Properties and Measurement Invariance of the Brief Symptom Inventory-18 Among Chinese Insurance Employees.* Frontiers in psychology, 2018. **9**: p. 519.
21. Xiao, S., *Theoretical basis and application in research of Social Support Rating Scale.* J Clin Psychiatry, 1994. **4**.
22. Li, X., et al., *Illness uncertainty, social support, and coping mode in hospitalized patients with systemic lupus erythematosus in a hospital in Shaanxi, China.* PloS one, 2019. **14**(2): p. e0211313.
23. Xiao, H., et al., *The Effects of Social Support on Sleep Quality of Medical Staff Treating Patients with Coronavirus Disease 2019 (COVID-19) in January and February 2020 in China.* Medical science monitor : international medical journal of experimental and clinical research, 2020. **26**: p. e923549.
24. Giglio, L.M.F., et al., *Development and use of a biological rhythm interview.* Journal of affective disorders, 2009. **118**(1-3): p. 161-165.
25. Allega, O.R., et al., *Performance of the biological rhythms interview for assessment in neuropsychiatry: An item response theory and actigraphy analysis.* Journal of affective disorders, 2018. **225**: p. 54-63.
26. Reyes, A.N., et al., *Biological rhythm and emotional and behavioral problems among schoolchildren in Southern Brazil.* Chronobiology international, 2019. **36**(3): p. 353-359.
27. Cho, C.-H., et al., *Validation of the Korean Version of the Biological Rhythms Interview of Assessment in Neuropsychiatry.* Psychiatry investigation, 2018. **15**(12): p. 1115-1120.
28. Bekalu, M.A. and S. Eggermont, *Determinants of HIV/AIDS-related information needs and media use: beyond individual-level factors.* Health Commun, 2013. **28**(6): p. 624-36.
29. Yu, N. and Z. Jiang, *Preference and Trust: An Investigation of Information Source of COVID-19 Among People Over 50 Years.* Asia Pac J Public Health, 2020: p. 1010539520956428.
30. Barrett, P., *Structural equation modelling: Adjudging model fit.* Personality and Individual Differences, 2007. **42**(5): p. 815-824.
31. Cheng, S.-T. and A.C.M. Chan, *Social support and self-rated health revisited: is there a gender difference in later life?* Social science & medicine (1982), 2006. **63**(1): p. 118-122.
32. Zhang, M., et al., *Prevalence of psychological distress and the effects of resilience and perceived social support among Chinese college students: Does gender make a difference?* Psychiatry research, 2018. **267**: p. 409-413.
33. Soman, S., et al., *Gender Differences in Perceived Social Support and Stressful Life Events in Depressed Patients.* East Asian archives of psychiatry : official journal of the Hong Kong College of Psychiatrists = Dong Ya jing shen ke xue zhi : Xianggang jing shen ke yi xue yuan qi kan, 2016. **26**(1): p. 22-29.
34. Silverstein, B., et al., *The gender difference in depressive prevalence is due to high prevalence of somatic depression among women who do not have depressed relatives.* Journal of affective disorders, 2017. **210**: p. 269-272.
35. Escalera, C., et al., *Social support as a moderator in the relationship between intrusive thoughts and anxiety among Spanish-speaking Latinas with breast cancer.* Psycho-oncology, 2019. **28**(9): p. 1819-1828.

36. Wang, X., et al., *Associations of psychological distress with positive psychological variables and activities of daily living among stroke patients: a cross-sectional study.* BMC psychiatry, 2019. **19**(1): p. 381.
37. Ramkisson, S., B.J. Pillay, and W. Sibanda, *Social support and coping in adults with type 2 diabetes.* African journal of primary health care & family medicine, 2017. **9**(1): p. e1-e8.
38. You, J. and Q. Lu, *Sources of social support and adjustment among Chinese cancer survivors: gender and age differences.* Support Care Cancer, 2014. **22**(3): p. 697-704.
39. Tang, W. and Q. Dai, *Depressive symptoms among first-year Chinese undergraduates: The roles of socio-demographics, coping style, and social support.* Psychiatry Res, 2018. **270**: p. 89-96.
40. Ma, D.Y., et al., *The correlation between perceived social support, cortisol and brain derived neurotrophic factor levels in healthy women.* Psychiatry Res, 2016. **239**: p. 149-53.
41. Koh, K.B., et al., *The relation between anger management style, mood and somatic symptoms in anxiety disorders and somatoform disorders.* Psychiatry Res, 2008. **160**(3): p. 372-9.
42. Lundh, L.G. and M. Simonsson-Sarnecki, *Alexithymia, emotion, and somatic complaints.* J Pers, 2001. **69**(3): p. 483-510.
43. Zhou, X., et al., *From culture to symptom: Testing a structural model of "Chinese somatization".* Transcult Psychiatry, 2016. **53**(1): p. 3-23.
44. Wu, Z., S.A. An, and S. An, *Fathers' Role in Chinese Children's Education*, in *Father Involvement in Young Children's Lives: A Global Analysis*, J. Pattnaik, Editor. 2013, Springer Netherlands: Dordrecht. p. 301-316.
45. Xiao, H., et al., *The Effects of Social Support on Sleep Quality of Medical Staff Treating Patients with Coronavirus Disease 2019 (COVID-19) in January and February 2020 in China.* Med Sci Monit, 2020. **26**: p. e923549.
46. Coles, M.E., J.R. Schubert, and J.A. Nota, *Sleep, Circadian Rhythms, and Anxious Traits.* Current psychiatry reports, 2015. **17**(9): p. 73.
47. Pereira-Morales, A.J., et al., *Anxiety symptomatology, sex and chronotype: The mediational effect of diurnal sleepiness.* Chronobiology international, 2018. **35**(10): p. 1354-1364.
48. Reniers, R.L.E.P., et al., *Risk Perception and Risk-Taking Behaviour during Adolescence: The Influence of Personality and Gender.* PloS one, 2016. **11**(4): p. e0153842.
49. Kim, Y., I. Park, and S. Kang, *Age and gender differences in health risk perception.* Central European journal of public health, 2018. **26**(1): p. 54-59.
50. Tanovic, E., D.G. Gee, and J. Joormann, *Intolerance of uncertainty: Neural and psychophysiological correlates of the perception of uncertainty as threatening.* Clinical psychology review, 2018. **60**: p. 87-99.
51. Chan, E.Y.Y., et al., *Knowledge, attitudes, and practices of Hong Kong population towards human A/H7N9 influenza pandemic preparedness, China, 2014.* BMC public health, 2015. **15**: p. 943.
52. The, L., *COVID-19: fighting panic with information.* Lancet (London, England), 2020. **395**(10224): p. 537-537.

 Click here to access/download;Figure;Figure_1.tiff

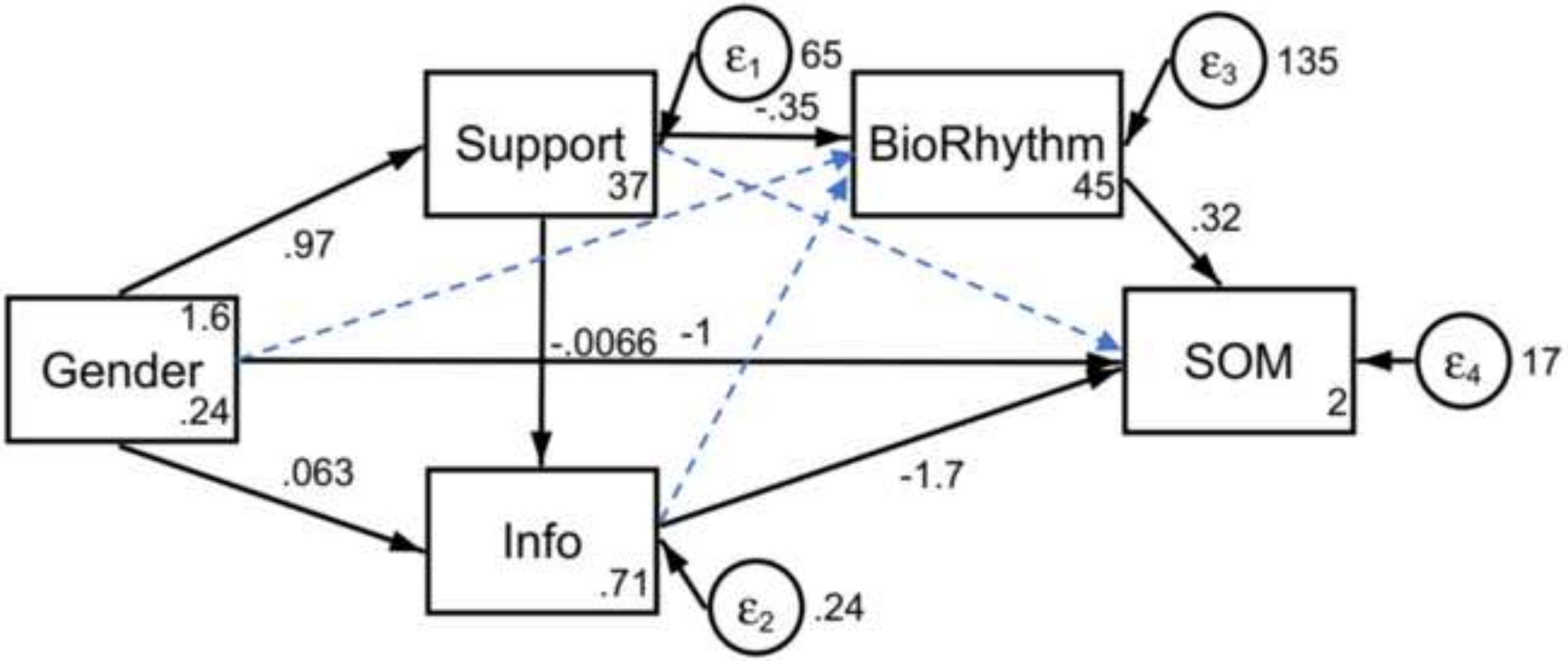

Chinese Version Inform Consent

Click here to access/download
**Supporting Information**
Inform Consent.pdf
