# Supplementary material for: Impact of the COVID-19 pandemic on psychological distress and biological rhythm in China’s general population: A path analysis model
Source: PLoS One. 2022 Jul 8;17(7):e0271285. doi: 10.1371/journal.pone.0271285 (PMC9269873; doi:10.1371/journal.pone.0271285)
Supplement: Attachment — Submitted filename: Response to Reviewers.docx [file pone.0271285.s004.docx]

Response to Editor and Reviewers

Dear editor. Yann Benetreau,

Re. Resubmission of PONE-D-21-14840, “Impact of the COVID-19 Pandemic on Psychological Distress and Biological Rhythm in China’s General Population: A Path Analysis Model”

Thank you for your action letter with the enclosed reviewers’ comments on our paper and your invitation to resubmit. We are grateful for the reviewers’ constructive comments. Our point-by-point response to the reviewers’ comments is immediately below this letter, and we have highlighted changes in red in the revised manuscript. We hope that by addressing the issues raised by the reviewers, the revised manuscript has been significantly improved and acceptable for publication in PLOS ONE. Should further revision be needed, please let us know.

Thank you very much for your time and consideration. We are looking forward to hearing from you.

Sincerely,

Xiaohong Ma

**Editor Comments:**

Requirements: When submitting your revision, we need you to address these additional requirements.

**(1) Please ensure that your manuscript meets PLOS ONE's style requirements, including those for file naming. The PLOS ONE style templates can be found at** [**https://journals.plos.org/plosone/s/file?id=wjVg/PLOSOne_formatting_sample_main_body.pdf**](https://journals.plos.org/plosone/s/file?id=wjVg/PLOSOne_formatting_sample_main_body.pdf) **and** [**https://journals.plos.org/plosone/s/file?id=ba62/PLOSOne_formatting_sample_title_authors_affiliations.pdf**](https://journals.plos.org/plosone/s/file?id=ba62/PLOSOne_formatting_sample_title_authors_affiliations.pdf)

**Response: Thank you for your suggestion. We have downloaded the PLOS ONE style templates and revised our manuscript according to the templates.

**(2) You indicated that you had ethical approval for your study. In your Methods section, please ensure you have also stated whether you obtained consent from parents or guardians of the minors included in the study or whether the research ethics committee or IRB specifically waived the need for their consent.**

**Response: Thank you for pointing out this problem. We have re-described the contents of our manuscript. (“Informed consent was acquired before each participant decided to finish this questionnaire. For juvenile participants, informed consent was obtained from their parents or guardians. To protect the privacy of participants, all collected information was anonymous.”)

**(3) Please complete all items on the Clinical Studies Checklist that are relevant for your submission, by following this link:**

[**http://journals.plos.org/plosone/s/file?id=dc11/PLOSOne_Clinical_Studies_Checklist.docx**](http://journals.plos.org/plosone/s/file?id=dc11/PLOSOne_Clinical_Studies_Checklist.docx)

**(Contact us at plosone@plos.org if you cannot access the document.)**

**There may be overlap between the checklist items and other queries listed below; please address any duplicated queries both in your response email and on the checklist itself. Upload the completed Clinical Studies Checklist as file type “Other” when you re-submit your manuscript. This document is for internal journal use only and will not be published if your article is accepted. The requested information will help us to assess whether your submission complies with PLOS ONE’s policies and adheres to applicable reporting standards.** **Note that your manuscript may be rejected if you provide incomplete or inadequate responses to the checklist questions and that changing the ‘Section/Category’ of your article does not affect this requirement.**

**Response: Thank you for your comments. We have uploaded the corresponding files. Please check these files for details.

**(4) Please change "female” or "male" to "woman” or "man" as appropriate, when used as a noun (see for instance** [**https://apastyle.apa.org/style-grammar-guidelines/bias-free-language/gender**](https://apastyle.apa.org/style-grammar-guidelines/bias-free-language/gender)**).**

**Response: Thank you very much. We have revised these words in our manuscript according to your suggestions. Please check revised manuscript for details.

**(5) Please include captions for your Supporting Information files at the end of your manuscript, and update any in-text citations to match accordingly. Please see our Supporting Information guidelines for more information:** [**http://journals.plos.org/plosone/s/supporting-information**](http://journals.plos.org/plosone/s/supporting-information)**.**

**Response: Thank you for your suggestion. We have added related contents in our manuscripts. Please check revised manuscript for details.

**(6) Please review your reference list to ensure that it is complete and correct. If you have cited papers that have been retracted, please include the rationale for doing so in the manuscript text, or remove these references and replace them with relevant current references. Any changes to the reference list should be mentioned in the rebuttal letter that accompanies your revised manuscript. If you need to cite a retracted article, indicate the article’s retracted status in the References list and also include a citation and full reference for the retraction notice.**

**Response: Thank you for your suggestion. We have carefully reviewed our references list and there are no incomplete and incorrect references in our list. Besides, according to the suggestions of reviewer #1, we have cited some new meaningful references and highlighted these new added references in red in revised manuscript.

**Review Comments：**

**Response to reviewer#1**

**(1) I have the following comments for the authors to address. I am happy to review this paper again***.*

**Response: Thank you very much for your comments and suggestions. Please see below for our point-by-point responses.

**(2) Under the Introduction, the authors stated "Social distancing, self-isolation and travel restrictions have led to a reduced workforce across all economic sectors and caused many jobs to be lost'". Please discuss the impact of social distancing, lockdown and facemask use on mental health:** **Social distancing on mental health: *Impact of COVID-19 on Economic Well-Being and Quality of Life of the Vietnamese During the National Social Distancing. Front Psychol. 2020 Sep 11;11:565153. doi: 10.3389/fpsyg.2020.565153. PMID: 33041928; PMCID: PMC7518066.* Lockdown on mental health: *Anxiety and Depression Among People Under the Nationwide Partial Lockdown in Vietnam. Front Public Health. 2020;8:589359. Published 2020 Oct 29. doi:10.3389/fpubh.2020.589359* Facemask on mental health: *The Association Between Physical and Mental Health and Face Mask Use During the COVID-19 Pandemic: A Comparison of Two Countries With Different Views and Practices. Front Psychiatry. 2020;11:569981. Published 2020 Sep 9. doi:10.3389/fpsyt.2020.5699812)***

**Response: We have stressed the impact of social distancing on economic well-being, and cited the recommended reference (“Social distancing, self-isolation, and travel restrictions have led to downsize or closure of businesses as well as a reduced workforce across all economic sectors, causing many job losses and family income losses[5]”). For the influence of lockdown on mental health, we have re-described the impact on those who lived alone and emphasized their psychological distress like anxiety, depression, etc. that they may have suffered from. The recommended reference has also been cited in this sentence. (“The elderly, the single, the separated and those who also live alone for various reasons have to face the horrible infectious disease without family members’ company, which has caused a rising level of mental issues like anxiety, stress, and depression among such special population [14].”). As for the impact of facemask on mental health, we have realized the importance of this factor and carefully read the recommended reference. Conflicting information for usage of facemask in anti-infection makes general population confused and concerned. (“For example, while facemask wearing is a positive precaution, it still goes through social stigma, arousing mixed opinion and contradictory messaging from the media, which all lead to public fear and confusion [17]”)

**(3) Under the Introduction, the authors stated "Approximately 10%-30% general population were much concerned with being infected when an influenza outbreak occurred [10]." It is more appropriate to discuss the impact on general population during the pandemic instead of influenza. The following is a multinational study and its finding worth mentioning: *The impact of COVID-19 pandemic on physical and mental health of Asians: A study of seven middle-income countries in Asia. PLoS One. 2021 Feb 11;16(2):e0246824. doi: 10.1371/journal.pone.0246824. PMID: 33571297.3)***

**Response: Thank you for pointing out this problem. We have added more information based on the background of pandemic instead of on influenza outbreak only, and the mentioned reference has been cited in the sentence below. (“Moreover, a survey base on seven middle income countries in the general population show high risk factors on mental health during the COVID-19 pandemic which relate to single or separated status, high educated level, age< 30 years [12].”)

**(4) Under the discussion, the authors stated "Nevertheless, most previous studies reported that females suffer from more somatization symptoms than males and are more vulnerable to psychological distress [32, 34]." Reference 32 and 34 are not from China. Please check with studies from China whether they report similar or different finding: *A Longitudinal Study on the Mental Health of General Population during the COVID-19 Epidemic in China [published online ahead of print, 2020 Apr 13]. Brain Behav Immun. 2020; S0889-1591(20)30511-0. doi:10.1016/j.bbi.2020.04.0284)***

**Response: Thank you for your comments. To our excited, we have found some similar results in the recommended references above. This longitudinal study in China showed that male gender might suffer from more psychological distress (except PTSD symptoms) than female gender both in first survey and second survey. And we have re-described these sentences in revised manuscript. (“In our model, being female was a protective factor and this result is inconsistent in different studies. Some extant studies showed that in women, the prevalence of psychological distress was higher and somatization symptoms were more obvious [12, 39]. While, a longitudinal study of general population in China during COVID-19 pandemic suggested that male gender showed higher association with stress, anxiety and depression than female gender [43].”)

**(5) Under the discussion, the authors should mention the relationship between physical or somatic symptoms and mental health based on the following study: *"A chain mediation model on COVID-19 symptoms and mental health outcomes in Americans, Asians and Europeans. Sci Rep 11, 6481 (2021). https://doi.org/10.1038/s41598-021-85943-7"5)***

Response: Thank you. We have read this meaningful reference carefully and added some the following sentences between the somatic symptoms and mental health. (“A chain mediation model study in Americans, Asians, and Europeans showed that seeking for health information served as a mediator between physical symptoms and the perceived influence of the COVID-19 pandemic. Overloading, conflicting, and ambiguous health information might increase burden of mental health [56].”)

**(6) Please discuss how to reduce somatic symptoms. I recommend Internet cognitive behavior therapy (iCBT) as it will reduce face to face contact and improve mental well being: The most evidence-based treatment is cognitive behaviour therapy (CBT), especially Internet CBT that can prevent the spread of infection during the pandemic. Please refer to the following studies: Use of Cognitive Behavior Therapy (CBT) to treat psychiatric symptoms during COVID-19:**

***Mental Health Strategies to Combat the Psychological Impact of COVID-19 Beyond Paranoia and Panic. Ann Acad Med Singapore. 2020;49(3):155‐160.***

***Cost-effectiveness of iCBT: Moodle: The cost effective solution for internet cognitive behavioral therapy (I-CBT) interventions. Technol Health Care. 2017;25(1):163-165. doi: 10.3233/THC-161261. PMID: 27689560.***

***Internet CBT can treat psychiatric symptoms such as insomnia: Efficacy of digital cognitive behavioural therapy for insomnia: a meta-analysis of randomised controlled trials. Sleep Med. 2020 Aug 26;75:315-325. doi: 10.1016/j.sleep.2020.08.020. Epub ahead of print. PMID: 32950013.6)***

**Response: Thank you for your suggestions. Internet cognitive behavioral therapy (I-CBT) is an effective measure to aid home-quarantine people who suffer from insomnia or other somatic symptoms. In the Discussion section, we have added more content related to I-CBT according to your recommended references. (“Biological rhythm disorder is a risk factor and needs to be intervened by professional psychologists or psychiatrists. Lockdown and home-quarantine restrict the access of psychological guidance. Hence, the internet cognitive behavioral therapy (i-CBT) as an effective measure can alleviate psychological distress and improve mental well-being, which is worthwhile to be implemented among those having insomnia or physical symptoms [53-55].”)

**(7) Please discuss how somatic symptoms may affect COVID-19 vaccine as people may think somatic symptoms as side effects of vaccines. Please comment on the following vaccine study and how somatic symptoms may affect attitude towards COVID-19 vaccine: *Attitudes toward COVID-19 vaccination and willingness to pay: Comparison of people with and without mental disorders in China. BJPsych Open, 7(5), E146. doi:10.1192/bjo.2021.979***

**Response: Thank you for your suggestion. Vaccine uptake is an important process for herd immunity. As you have emphasized it indeed related with some physical symptoms. Misleading information exaggerates the fear and concern. And we have referred to the article mentioned above that people with mental disorder show higher acceptance of vaccine. (“Besides, the acceptance of COVID-19 vaccine is also affected by misleading, contradictory media information. To some extent, hesitation in front of vaccine can be increased by rumors and stigmas. COVID-19 related somatic symptoms and other psychological distress were associated with higher willingness of vaccine uptake [60]. In particular, patients with mental illness have a higher vaccine acceptance [61, 62]”)

**Response to reviewer#2**

Dear reviewer #2,

Thank you for your comments and revision of the language and vocabulary of our manuscript. We have read the comments in email and attachment PDF file carefully and prepared one-by-one replies as listed below.

**(1) The article is well-written and provides some interesting insight on the psychological impact of COVID-19, with a particular attention on gender differences. The references are provided to a satisfying extent. Finally, the findings support the authors' conclusions and the sample size is totally reliable.**

**Response: Thank you for your positive comments regarding our paper.

**(2) I just have a few concerns as regards the SEM/Path analysis.** **I wasn't able to grasp whether the analysis at hand is a proper SEM (therefore, with at least 1 latent variable) or a mere path analysis wherein all the variables are observed and we can assume no error. There seems to be some confusion about this aspect and I would definitely recommend further specifications on this issue. In my opinion, this is the most critical point of the article; thus, it should be taken in serious consideration by the authors.**

**Response: Thank you for bringing about this important issue. Path analysis is a special case of SEM. It contains only observed variables, and has a more restrictive set of assumptions than SEM. Most of the models we can see in the literature are SEM rather than path analyses. The main difference between the two types of models is that path analysis assumes that all variables are measured without error. SEM uses latent variables to account for measurement error. Path analysis is a subset of Structural Equation Modeling (SEM), a multivariate procedure.

The complete structural equation modeling includes both the measurement model and the structural model, and the absence of latent variables means that there is no measurement model, only the structural model. Therefore, it should be said that path analysis is a structural equation modeling with only structural models.

In a review about application of SEM in psychological research described as follows: “Some special cases of SEM are of particular interest and are commonly used in practice. For example, path analysis provides for the testing of models of relationships among measurement variables (MVs); no latent variables(LVs) are included in the model. Path analysis models are used when MVs are of primary interest or when multiple indicators of LVs are not available. Factor analysis, another special case, provides for testing models of relationships between LVs, which are common factors, and MVs, which are indicators of common factors. The factor analysis model allows for correlational (nondirectional) relationships among LVs but does not include directional influences as in general SEM.”(MacCallum, R. C., & Austin, J. T. (2000). Applications of structural equation modeling in psychological research. Annual Review of Psychology, 51, 201–226. https://doi.org/10.1146/annurev.psych.51.1.201) And we have drawn a schematic diagram about the relationship of different statistical models (Beaujean, A. A. . (2014). Latent Variable Modeling Using R. New York: Routledge.). We hope this clarification helps.

**Figure 1** (in letter) Families of Statistical Models

**(3) One last point: I am wondering whether the authors have considered to perform a multigroup SEM to see how the paths work for the two genders separately; i.e., whether some of the paths are significantly stronger for one of the genders.**

**Response: Thank you for your suggestion. We have performed a multigroup SEM, and results are as follows:

**Table 1** (in letter) Fit indices of multigroup SEM between gender

|  | **x^2^** | **P Value*** | **RMSEA*** | **CFI*** | **TLI*** | **SRMR*** |
| --- | --- | --- | --- | --- | --- | --- |
| **Path Analysis Model** | 1.55 | 0.817 | 0.0000 | 1.0000 | 1.0000 | 0.005 |

**(a)Men	(b)Women**

**Figure 2**(in letter) The estimated paths in **(a)** men and **(b)** women

**Table 2** (in letter) Unstandardized direct and indirect effects of study variables

|  | **Men** | | **Women** | | |  |
| --- | --- | --- | --- | --- | --- | --- |
|  | **Direct Effects** | **Indirect Effects** | | **Direct Effects** | **Indirect Effects** | |
| **Support→Info** | -0.0024 | - | | -0.0097** | - | |
| **Support→BioRhythm** | -0.2626** | - | | -0.4175** | - | |
| **BioRhythm→SOM** | 0.3654** | - | | 0.2785** | - | |
| **Info→SOM** | -1.5789** | - | | -1.6713** | - | |
| **Support→SOM** | - | -0.0922** | | - | -0.1001** | |

Note: *: P < 0.05，**: P < 0.001

**(4) I hereby attach a pdf file with 19 comments on several other points of the article.**

**Response: Thank you for your comments. We have listed these comments below. Please check the next replies for details.

**(5) Did the author want to say "intimate"?**

**Response: Yes, this word has been revised in the manuscript. (“In many countries and regions, governments asked residents to reduce unnecessary outdoor activities, and shopping malls and public transportations were also closed to avoid intimate contact.”)

**(6) Oxford comma is missing before "and"**

**Response: Revised as suggested. (“Social distancing, self-isolation, and travel restrictions have led to downsize or closure of businesses as well as a reduced workforce across all economic sectors, causing many job losses and family income losses[5].”)

**(7) Missing comma before "etc."**

**Response: Revised as suggested. (“Staying up late, getting up late, and lying in bed during non-sleeping time all decrease activity and meal frequency, causing rhythm disorders in eating, sleeping, social activities, etc. and aggravating people's physical and psychological distress in the meantime.”)

**(8) I see there is an ID for the Ethics Committe's approval: No.2020-178. Please report it in the main text.**

**Response: Done as suggested. (“This research was approved by the Ethics Committee of West China Hospital of Sichuan University (No.2020-178).”)

**(9) Please use the plural "instruments"**

**Response: Revised as suggested. (“**Measure Instruments”**)

**(10) It is unclear what comparisons were used by the authors here, via the t-tests. Please specify. Was gender the factor (IV)?**

**Response: Revised as suggested. (“First, for continuous variables such as age and scores of psychological distress, *t* test was used to assess the statistical significance between men and women”)

**(11) Same comment here. Was it the distribution of education level within the two genders? Please specify.**

**Response: Thank you for your comment. We have revised in the manuscript. (“For categorical variables between men and women, x^2^ test was used to describe the constituent ratio of education level and media information preference, etc.”)

**(12) The authors indifferently refer to their analysis as a SEM model and a path analysis. Unfortunately, in this sentence, two levels are mixed together: one is the level of how the variables are connected (i.e., outcome variable, input variable, mediator, eventual moderator, etc.); the other one is the level of how the variables are modeled (observed, latent).For the analysis to be a proper SEM, at least a latent variable should be present. Is that the case? The authors never mention eventual parcels of latent variables; therefore, this should be taken into serious consideration: if no latent variable is present, then it is uncorrect to refer to this analysis as a SEM model. The authors should definitely clarify this point.**

**Response: Thank you for your valuable comments. Please see our response about comment #2 (Page5-6).

**(13) Please note that this statement is not true for SEM! On the contrary, Chi-square statistics in SEM test the null hypothesis that the Model fits the data perfectly (i.e., predicted model and observed data are equal) and a value of P > 0.05 (fail to reject null hypothesis) is recommended.**

**Response: We indeed made a mistake in writing “P < 0.05”. Sorry for the typo. As you have noted, it would be a perfect model if P > 0.05. **We meant to say that except chi-square, more suitable indices could also be considered in a path analysis model.** Some researchers disregard this index if both the sample size exceeds 200 or so and other indices indicate the model is acceptable. In particular, this approach arises because the chi-square index presents several problems: 1) Complex models, with many parameters, will tend to generate an acceptable fit; 2) If the sample size is large, the model will usually be rejected, sometimes unfairly.

**(14) Please provide some reference for these thresholds**

**Response: Done as suggested. (“It would be considered as a good model if RMSEA <0.08, SRMR <0.08, and GFI >0.90[34-36].”)

**(15) As for the categorical variables, the reader would greatly benefit from the percentage within each gender. For instance, Male Urban areas 768 (60.14%), Rural areas 509 (39.86%), etc.**

**Response: Thank you for your suggestion. We have added the percentage information in Table 1 for categorical variables.

**Table 1**(in manuscript) Gender Difference in Social-demographic Information, Psychological Distress, Social Support,

Biological Rhythm, and Media Information Preference

|  | Men (N=1277) | Women (N=1960) | T/X^2^ Value | P Value |
| --- | --- | --- | --- | --- |
| ***Age*** | 30.70±9.36 | 31.12±9.56 | -1.23 | 0.2162 |
| ***Education Level*** |  |  |  |  |
| Middle School or Below | 122 (9.55%) | 136 (6.94%) | 7.58 | 0.0560 |
| High School | 148 (11.59%) | 221 (11.27%) |  |  |
| Bachelor’s degree | 771 (60.38%) | 1221 (62.30%) |  |  |
| Master’s degree or Above | 236 (18.48%) | 382 (19.49%) |  |  |
| ***Residence*** |  |  |  |  |
| Urban Areas | 768 (60.14%) | 1268 (64.69%) | 6.87 | 0.0090 |
| Rural Areas | 509 (39.86%) | 692 (35.31%) |  |  |
| ***Marital Status*** |  |  |  |  |
| Unmarried | 661 (51.76%) | 911 (46.48%) | 8.64 | 0.0030 |
| Married | 616 (48.24%) | 1049 (53.52%) |  |  |
| ***Information Preference*** |  |  |  |  |
| Positive or Negative Info. | 614 (48.08%) | 832 (42.45%) | 9.92 | 0.0020 |
| Both of the Above | 663 (51.92%) | 1128 (57.55%) |  |  |
| ***Social Support Total Scores*** | 37.57±8,26 | 38.54±7.89 | -3.36 | 0.0008 |
| ***Somatization Scores*** | 9.98±6.21 | 9.13±5.19 | 4.20 | <0.0001 |
| ***Depression Scores*** | 10.84±6.34 | 10.44±5.62 | 1.88 | 0.0606 |
| ***Anxiety Scores*** | 10.4±6.29 | 10.06±5.71 | 1.57 | 0.1170 |
| ***Global Severity Index*** | 31.22±18.37 | 29.64±15.66 | 2.62 | 0.0088 |
| ***BRIAN Total Scores*** | 31.27±12.58 | 32.07±11.54 | -1.86 | 0.0633 |

**(16) In the light of possible future metanalyses, could the authors provide the effect size for such differences?**

**Response: Thanks. We make a supplementary table for variables’ effect size in our manuscript as follows.

**S1 Table.** (in manuscript) Effect size of social-demographic information, psychological distress, social support, biological rhythm, and media information preference

|  | Cohen's d/  Cramer's Phi* | 95% Confidence Interval |
| --- | --- | --- |
| ***Age*** | -0.0445 | -0.1150~0.0260 |
| ***Education Level*** |  |  |
| Middle School or Below | 0.0484 | 0.0133~0.0757 |
| High School |  |  |
| Bachelor’s degree |  |  |
| Master’s degree or Above |  |  |
| ***Residence*** |  |  |
| Urban Areas | -0.0461 | -0.0807~-0.0114 |
| Rural Areas |  |  |
| ***Marital Status*** |  |  |
| Unmarried | 0.0517 | 0.0170~0.0862 |
| Married |  |  |
| ***Information Preference*** |  |  |
| Positive or Negative Info. | 0.0554 | 0.0211~0.0897 |
| Both of the Above |  |  |
| ***Social Support Total Scores*** | -0.1208 | -0.1914~-0.0503 |
| ***Somatization Scores*** | 0.1509 | 0.0803~0.2215 |
| ***Depression Scores*** | 0.0675 | -0.0030~0.1380 |
| ***Anxiety Scores*** | 0.0564 | -0.0141~0.1269 |
| ***Global Severity Index*** | 0.0942 | 0.0237~0.1648 |
| ***BRIAN Total Scores*** | -0.0668 | -.0.1373~0.0037 |

Note: * For continuous variables like age, social support total scores and other scales’ scores, we used Cohen's d to show the effect size. For categorical variables like education level, residence, marital status, and information preference, we used Cramer's Phi to show the effect size.

**(17) Each time that gender is used as a dichotomous variable, the authors should mention whether the major value stands for male or female.**

**Response: Done as suggested. (“Obviously, being female had a positive correlation with media information preference (r=0.0554), social support scores (r=0.0546), and BRIAN total scores (r=0.0517). However, being female had a negative correlation with somatization scores (r=-.00380).”)

**(18) Typo**

**Response: Revised in the manuscript. (According to correlations among study variables, we built a SEM to explore the possible causal relationship among the study variables, and the fit indices were depicted.)

**(19) are the β standardized or not? Please specify.**

**Response: Thank you for your comment. These β are unstandardized coefficients in our manuscript. We have noted them in result section (“Women were associated with a higher level of social support (unstandardized β=0.9715, P=0.001) and lower somatization scores of psychological distress (β=-1.0007, P<0.001). Men (unstandardized β=0.0628, P<0.001) were associated with more extreme media information.”) And for β in Table 4 and Table 5 have also been noted. ( Please see Table 4 & Table5 for details)

**(20) Are those 90 or 95% CI? Please specify.**

**Response: Thank you for pointing out this problem. These confidence intervals are 95% and we have revised accordingly in Table 4 and Table 5. (Please see Table 4 & Table5 for details)

**(21) Please use the plural**

**Response: Revised in the manuscript. (“In this cross-sectional study involving 3,237 participants, we found significant differences in social total scores and global severity index, which goes in line with the existing literature.”)

**(22) Please add some references for these studies**

**Response: Revised in the manuscript. (“These results have been partly confirmed by previous studies[18, 20]”)

**(23) Please correct the typo**

**Response: Revised in the manuscript. (“The study is the first step to uncover the direct and indirect effects of gender on somatization symptoms of psychological distress during the COVID-19 pandemic, while social support, biological rhythm, and information preference can be used as possible mediators.”)
