# Supplementary material for: Impact of the COVID-19 pandemic on psychological distress and biological rhythm in China’s general population: A path analysis model
Source: PLoS One. 2022 Jul 8;17(7):e0271285. doi: 10.1371/journal.pone.0271285 (PMC9269873; doi:10.1371/journal.pone.0271285)
Supplement: Attachment — Submitted filename: Response to Reviewers.docx [file pone.0271285.s005.docx]

**Responses to Editors and Reviewers**

| **Editors Comments** | **Author responses** |
| --- | --- |
| Thank you for your revision. One of the reviewers found minor issues that need to be fixed before the paper is published. Please address them and resubmit. | Thank you very much. We have tried to fix these issues as suggested. Please check our revised manuscript for details.  Besides, we have something else to report to the editors.  1. In the original Introduction, we cited some data (“Exceeding 153,000,000 people were confirmed to be infected with this severe respiratory infectious disease, and more than 216 countries or territories were affected [2]”) to show the severe impact of COVID-19 pandemic on the whole world. Nevertheless, this figure (153,000,000) is out of date now and the latest data retrieved from WHO’s official website says 513,955,910. We therefore revised the sentence as follows and renewed the references: “The latest data form WHO’s official website showed that this severe respiratory infectious disease has infected more than 500 million people and caused over 6 million to die (by early May 2022).”) (*line 40-42*)  2. Two references were removed in the revised manuscript. One has been explained above; and the other has been deleted according to Reviewer #3’s suggestion. Please check our response to reviewer Point #3, Page 2 in this letter for details.  3. Figure 1 has been adjusted and re-uploaded in the submission system, since we were suggested to report standardized coefficients in the revised manuscript. |
| **Reviewer #3 comments** | **Author responses** |
| The authors sought to assess the influence of gender on social support, information preference, biological rhythm, and psychological distress during the COVID-19 pandemic. This addresses the gap of limited studies on the influence of gender on psychosomatic outcomes in the context of the COVID-19 pandemic. The authors used path analysis to assess the primary outcome of somatization. Overall, they found gender differences in factors predicting somatization and the important role of social support as a protective factor. Because of the paucity of literature on the influence of gender on somatization in the Chinese population in the context of COVID-19, this study helps to address this literature gap. | Thank you for your positive comments regarding our paper. |
| 1. This is a sound paper however there are numerous grammatical errors and areas where the authors sometimes switch between tenses (i.e. switch between present tense and past tense) that will need to be addressed. | We apologize for the grammatical errors that still persist. We have read the comments carefully and prepared one-by-one replies as listed below. And we hope this version is better and has addressed your concern regarding language. All the changes were marked in red in *Revised* *Manuscript with Track Changes*. |
| 2. Standardized coefficients are not presented. | Thank you for your suggestion. For coefficients in Table 4, Table 5 and main body, we have changed it to standardized value. Please see *line 209, 210, 217, 226 ,227, 232, 233, 236, 243* and *244* in our *Revised* *Manuscript with Track Changes* for details. (Revised Table 4 and Table 5 have been placed at the end of this letter. ) |
| **Introduction**  3. Since you cited Chinese studies, the sentence on influenza is not needed: “Approximately 10%-30% general population were much concerned with being infected when there was an influenza outbreak[11]” | Thank you. We have removed the sentence and reference as suggested. |
| 4. Typos in this sentence: “Moreover, a survey based on seven middle income countries in the general population showed high risk factors on mental health during the COVID-19 pandemic which related to single or separated status, high education level, and age< 30 years [12].” | Revision: “Moreover, a survey based on seven middle income countries in the general population showed high risk factors on mental health during the COVID-19 pandemic which were related to single or separated status, high educated level, and age < 30 years.” (*line 52-55*) |
| 5. This sentence is confusing: “Furthermore, social media of unreliable sources usually provide much ambiguous epidemic information, and overloaded information may cause psychological distress in turn [16]. For example, while facemask wearing is a positive precaution, it still goes through social stigma, arousing mixed opinion and contradictory messaging from the media, which all lead to public fear and confusion [17].” Should it read like this instead? “Furthermore, social media using unreliable sources, usually provide ambiguous epidemic information, and information overload may cause psychological distress in turn [16]. For example, while facemask wearing is a positive precaution, its use brings about social stigma, arousing mixed opinion and contradictory messaging from the media, which all lead to public fear and confusion [17].” Please clarify. | Thank you for your comment. Sorry for the confusion. We have revised as you suggested. (“Furthermore, social media using unreliable sources usually provide ambiguous epidemic information, and information overload may cause psychological distress in turn [14]. For example, while facemask wearing is a positive precaution, its use brings about social stigma, arousing mixed opinion and contradictory messaging from the media, which all lead to public fear and confusion[15].”) (*line 64-67*) |
| 6. Remove “etc” from the sentence: “Staying up late, getting up late, and lying in bed during non-sleeping …, social activities, etc. and aggravating people's physical and psychological distress in the meantime.” | Done as suggested. (“Staying up late, getting up late, and lying in bed during non-sleeping time all decrease activity and meal frequency, causing rhythm disorders in eating, sleeping, social activities, and aggravating people's physical and psychological distress in the meantime.”) (*line 77*) |
| **Methods**  7. Typos with APPS (make it lowercase) and ‘research’ is plural: “2) Both negative and positive media information. This question was designed based on some previous researches which aimed to reflect the preference of different respondents for the magnanimity of media information on cellphone social apps or television [16, 20, 32, 33].” | Thank you very much. We have revised these words in our manuscript according to your suggestions. Please see *line 86*, *133* and *134* in our *Revised* *Manuscript with Track Changes* for details. |
| 8. The authors mention aiming to study possible causal relationships. However, causality cannot be assessed in a cross-sectional study. Please revise. Also correction with grammar: “Finally, aiming to study the possible causal relationship…path analysis to test the relationship among interrelated study variables in a hypothesized model.” | Thank you for pointing out this problem. We have revised accordingly. (“Finally, aiming to explore the overall relationship among multiple variables, we constructed a structural equation model (SEM) and applied path analysis to test the relationship among interrelated study variables in a hypothesized model.”) (*line 144-146*) |
| **Results**  9. For Chi square the authors use both lower “x2” and upper case “X2”. Please be consistent and use upper case X2 throughout manuscript. Similar suggestion for “p values”. Use P or p to match how it is recommended by the journal. ‘p’ for values of p<0.001. | Thank you for your suggestions. We have uniformly used upper case “X^2^” in our paper. Please see *line 140, 167, 169, 177* and *202* in *Revised* *Manuscript with Track Changes* for details.  Similarly, we have revised “P” to lower case “p” in our article. Please see *line* *152,159, 168, 172, 173, 177, 191, 199, 202, 204, 209, 219, 226, 227, 232, 233, 236* and *243* in *Revised* *Manuscript with Track Changes* for details. |
| 10. Correction: (S1 Table shows the effect size of study variables…) | Thank you. We have corrected as suggested. (“S1 Table shows the effect size of studying variables in Table 1”) (*line 174*) |
| 11. The authors go back and forth between using “men”, “women”, “males”, and “females”. Please be consistent and use terms appropriately. | Thanks for raising this important issue. We have revised these words and uniformly used “men” and “women”. Please see *line 180, 182, 259, 262* and *263* in *Revised* *Manuscript with Track Changes* for details. |
| 12. It is suggested to remove the word “obviously” from the manuscript. | Done as suggested. Please see *line 180* in *Revised* *Manuscript with Track Changes* for details. |
| 13. Again, the mention of exploring causality is mentioned and is not possible for a cross-sectional study. | Thank you for your valuable comments. We have revised it as follows. (“According to correlations among study variables, we built a SEM to explore the overall relationship among the multiple study variables, and the fit indices were depicted (Table 3).”) (*line 194-195*) |
| 14. Please report standardized coefficients and R^2^ values for the final model in table. | For standardized coefficients please see our response to Point #2, Page 2 in this letter.  Meanwhile, we have added R^2^ values for the final model behind related model fit indices in figure legend (*line 221*). Please check it in our *Revised* *Manuscript with Track Changes* for details. |
| **Discussion**  15. Did the authors mean “In this cross-sectional study involving 3,237 participants, we found significant differences in social support total scores and…”? | Sorry for the ambiguity. We wanted to stress differences between men and women in scores of SSRS and BSI-18. We have re-described the contents of our manuscript. (“In this cross-sectional study involving 3,237 participants, we found significant differences in social support total scores and global severity index between men and women, which goes in line with the existing literature.”) (*line 247-249*) |
| 16. Again, the authors switch between using “men”, “women”, “males”, and “females”. Please address this. | Revised as suggested. Please see our response to Results Point #11, Page 4 in this letter. |
| 17. Correction: “Third, for unmarried or single men who lived alone, self-isolation may be a big challenge…be confused by epidemic information and experience increased fear of the COVID-19 pandemic.” | Thank you. We have corrected as suggested: (“Third, for unmarried or single men who lived alone, self-isolation may be a big challenge because of insufficient social support and limited ways of expression. They may easily be confused by epidemic information and experience increased fear of COVID-19 pandemic.”) (*line 278*) |
| 18. Correction: “Besides, the acceptance of the COVID-19 vaccine is also affected by…” | Done as suggested. (“Besides, the acceptance of COVID-19 vaccine was also affected by misleading, contradictory media information.”) (*line 314*) |
| 19. This sentence is confusing: “To some extent, hesitation in front of vaccine can be increased by rumors and stigmas.” Are the authors trying to say that rumors and stigma increase an individual’s hesitancy for vaccination uptake? Please reword for clarity. | Sorry for the confusion. We have reworded as suggested. (“To some extent, rumors and stigma for vaccine increase an individual’s hesitancy for vaccination uptake.”) (*line 315-316*) |
| 20. Please use correct in-text citation based on journal style to cite: “Therefore, as Wong L P emphasized, the dissemination of epidemic information…” | Thank you for your suggestion. To be more formal, we removed the author's name in this sentence and replaced it with “a previous study”. (“Therefore, as emphasized in previous studies, the dissemination of epidemic information should be dedicated to meeting the information needs of diverse sociodemographic and ethnic groups.”) (*line 320*) |

**Table 4**. Direct effects in Model 4

| **Path** | **Coefficient*** | **SE** | **Z value** | **p value** | **95% CI** |
| --- | --- | --- | --- | --- | --- |
| **Gender→Support** | 0.0590 | 0.0175 | 3.37 | <0.001 | 0.0247~0.0933 |
| **Gender→Info** | 0.0617 | 0.0174 | 3.54 | <0.001 | 0.0276~0.0959 |
| **Support→Info** | -0.1075 | 0.0174 | -6.19 | <0.001 | -0.1416~-0.0735 |
| **Gender****→****BioRhythm** | No Path | | | | |
| **Info→BioRhythm** | 0.0002 | 0.0172 | 0.01 | 0.9920 | -0.0335~0.0338 |
| **Support→BioRhythm** | -0.2348 | 0.0167 | -14.06 | <0.001 | -0.2675~-0.2020 |
| **Gender→SOM** | -0.0865 | 0.0127 | -6.82 | <0.001 | -0.1114~-0.0616 |
| **BioRhythm→SOM** | 0.6685 | 0.0101 | 66.16 | <0.001 | 0.6487~0.6884 |
| **Info→SOM** | -0.1468 | 0.0128 | -11.47 | <0.001 | -0.1718~-0.1217 |
| **Support→SOM** | -0.0095 | 0.0134 | -0.71 | 0.4794 | -0.0358~0.0168 |

Note: * standardized coefficients

**Table 5**. Direct effects and indirect effects in modified model

| **Path** | **Direct Effects** | | | | |  | **Indirect Effects** | | | | |
| --- | --- | --- | --- | --- | --- | --- | --- | --- | --- | --- | --- |
|  | **Coefficient*** | **SE** | **Z value** | **p Value** | **95% CI** |  | **Coefficient*** | **SE** | **Z value** | **p Value** | **95% CI** |
| **Gender→Support** | 0.0590 | 0.0175 | 3.36 | 0.001 | 0.2467~0.0933 |  | **No Path** | | | | |
| **Gender→Info** | 0.0617 | 0.0174 | 3.53 | <0.001 | 0.0276~0.0959 |  | -0.0063 | 0.0021 | -2.95 | 0.003 | -0.0105~-0.0021 |
| **Support→Info** | -0.1075 | 0.0174 | -6.15 | <0.001 | -0.1416~-0.0735 |  | **No Path** | | | | |
| **Gender→BioRhythm** | **No Path** | | | | |  | -0.0138 | 0.0042 | -3.26 | 0.001 | -0.0221~-0.0055 |
| **Support→BioRhythm** | -0.2348 | 0.0166 | -13.89 | <0.001 | -0.2673~-0.2022 |  | **No Path** | | | | |
| **Gender→SOM** | -0.0872 | 0.0127 | -6.85 | <0.001 | -0.1120~-0.0624 |  | -0.0174 | 0.0037 | -4.70 | <0.001 | -0.0247~-0.0101 |
| **BioRhythm→SOM** | 0.6707 | 0.0095 | 52.73 | <0.001 | 0.6521~0.6894 |  | **No Path** | | | | |
| **Info→SOM** | -0.1458 | 0.0127 | -11.45 | <0.001 | -0.1707~-0.1209 |  | **No Path** | | | | |
| **Support→SOM** | **No Path** | | | | |  | -0.1418 | 0.0122 | -11.64 | <0.001 | -0.1657~-0.1179 |

Note: * standardized coefficients
